# TangNaikang Formula Alleviates Podocyte Injury in Diabetic Nephropathy by Modulating the SHIP2/PI3K/AKT Pathway

**DOI:** 10.1155/jdr/6568591

**Published:** 2025-11-19

**Authors:** Tao Yang, Yongxin Huang, Wenjing Li, Lingling Qin, You Wu, Lili Wu, Tonghua Liu

**Affiliations:** ^1^Dongfang Hospital, Beijing University of Chinese Medicine, Beijing, China; ^2^Key Laboratory of Health Cultivation of the Ministry of Education, Beijing University of Chinese Medicine, Beijing, China; ^3^Ningbo Hospital of Traditional Chinese Medicine, Zhejiang Chinese Medical University, Ningbo, China

**Keywords:** diabetic nephropathy, PI3K/AKT pathway, podocyte injury, SHIP2, TangNaikang

## Abstract

**Aim:**

This study utilized db/db mice and MPC5 cells induced by high glucose as experimental models to examine the protective mechanisms of the traditional Chinese medicine formula TangNaikang (TNK) in mitigating podocyte injury in diabetic nephropathy (DN).

**Methods:**

The chemical constituents of TNK and TNK-containing serum were identified through UPLC-Q-TOF/MS. The underlying mechanism of TNK in treating DN was analyzed using network pharmacology. In vivo, following an 8-week intervention, db/db mice's serum biomarkers (TC, TG, HDL, LDL, AGEs, BUN, Scr, and *β*2-MG) were compared. H&E, PAS staining, and electron microscopy were used to perform a histopathological investigation on kidney sections. High glucose-induced MPC5 cells were treated with TNK-containing serum. Cellular viability was measured through a CCK-8 assay. The expression levels of podocyte-associated and PI3K/AKT pathway proteins in kidney tissues and MPC5 cells were determined by immunofluorescence, western blotting, and RT-qPCR analysis.

**Results:**

The UPLC-Q-TOF/MS results showed that the TNK formula consisted of 69 compounds, including flavonoids, triterpenoids, and lignans. TNK-containing serum was identified with 34 compounds including 9 TNK prototype components and 25 metabolites. TNK was found to be substantially linked with the PI3K/AKT pathway using network pharmacology. When compared to the model group, the TNK-H group mice had significantly improved serum lipid profiles as well as renal structural and functional profiles. Immunofluorescence and western blotting analyses indicated that TNK regulated the expression levels of the podocyte-associated (SYNPO, nephrin, CD2AP, and podocin) as well as PI3K/AKT pathway proteins (PI3K, AKT, SHIP2, IRS2, and GLUT4). These data were confirmed by RT-qPCR results. TNK-containing serum enhanced MPC5 cell viability via modulating the PI3K/AKT pathway and inhibiting SHIP2.

**Conclusion:**

TNK ameliorates podocyte injury in DN and high glucose-induced MPC5 cells by modulating the SHIP2/PI3K/AKT pathway.

## 1. Introduction

The International Diabetes Federation's most recent report in 2021 revealed that around 537 million persons globally are diabetic, with projections indicating that these numbers would increase to 783 million by 2045, particularly with higher incidence rates in China, India, and Pakistan [1]. Diabetic angiopathy is the most serious complication of diabetes and includes microvascular diseases affecting retinal capillaries and small arteries (retinopathy), nerves (neuropathy), and kidneys (nephropathy), as well as macrovascular diseases affecting the arteries, heart, and lower limbs [1, 2]. Over 700 million individuals globally are affected by chronic kidney disease (CKD). Diabetic nephropathy (DN) constitutes a primary etiology of CKD, while India and China represent approximately one-third of the prevalence and mortality associated with CKD [3, 4].

DN manifests through progressive elevation of urinary albumin excretion (UAE), heightened susceptibility to hypertension and cardiovascular disorders, declining estimated glomerular filtration rate (eGFR), and progression to end-stage renal disease [5]. A persistent hyperglycemic environment in individuals with diabetes negatively impacts glomerular podocytes, mesangial and endothelial cells, renal tubular epithelial cells, interstitial fibroblasts, and vascular endothelial cells [6–8]. The glomerular pathology in DN demonstrates prominent features such as mesangial cellular proliferation with hypertrophy, abnormal accumulation of extracellular matrix (ECM) components causing matrix expansion, thickened glomerular basement membranes (GBMs), and formation of nodular sclerotic lesions known as Kimmelstiel–Wilson nodules [9]. Podocyte injury represents a central pathogenic mechanism underlying proteinuria development in DN. Podocytes are terminally developed cells that reside on the outer surface of the GBM, along with vascular endothelial cells and the GBM, making up the glomerular blood filtration barrier. Therefore, podocytes are important for preserving the glomerular filtration membrane's structural integrity and function [10]. Podocyte injury is manifested through morphological and functional abnormalities, including abnormal structural and phenotypic changes in podocyte-related proteins as well as podocyte widening, fusion, and hypertrophy. This decreases the relative density of podocytes and disrupts the normal structure of the filtration membrane. Metabolic alterations in DN cause podocyte injury, apoptosis, and detachment in diabetes patients, leading to proteinuria and ESRD [11]. Currently, the clinical management of DN patients involves regulation of glucose levels and blood pressure through dietary and lifestyle interventions or drugs [12]. Research studies have indicated prevention of podocyte injury or stimulation of repair and regeneration of injured podocytes may be effective in alleviating DN.

SHIP2, discovered by Pesesse et al. in 1997, is an inositol polyphosphate 5-phosphatase with a relative molecular mass of 142 kD. Hyvönen et al. found that suppressing the overexpression of endogenous liver-specific SHIP2 could improve glucose metabolism and insulin resistance in db/db mice [13]. SHIP2 is a negative regulator of the insulin signaling pathway and plays an important role in podocyte damage and DN, which can specifically downregulate insulin signaling via the PI3K/AKT pathway by modulating its 5⁣′-phosphatase activity [14–19]. The glomerular filtration barrier, which prevents filtration of large plasma molecules, is a special protein complex connecting the foot processes of adjacent podocytes and includes proteins such as nephrin, actin, podocin, NEPH1, ZO-1, FAT, and P-calmodulin. Venkatareddy et al. reported that activated nephrin recruited and regulated a protein complex made up of SHIP2, filamin, and lamellipodin, which played an important role in regulating podocyte actin and focal adhesion dynamics, and lamellipodia formation [20]. The podocyte slit diaphragm protein CD2AP negatively regulates the insulin signaling pathway by binding to SHIP2 and contributes to metabolic disorders [21].

Traditional Chinese medicine (TCM) strategies for treating DN have shown tremendous promise in recent years due to its multipathway and multitarget effects. Tang et al. performed a meta-analysis of clinical trials and animal studies regarding the treatment of DN using TCM and indicated that the bioactive components of Chinese herbs can modulate glucose and lipid metabolism while providing protective effects on podocytes through antioxidant, anti-inflammatory, and antifibrotic mechanisms [22]. He et al. demonstrated that ginsenoside Rb1, a significant active component of *Panax notoginseng*, reduced cellular apoptosis and mitochondrial damage caused by high glucose levels, as well as improved renal parameters in DN models by lowering apoptotic protein expression [23]. Zhu et al. demonstrated that Zuogui pill, a classic Chinese medicine prescription for nourishing kidney yin, protected the kidneys and podocytes of the db/db mice by suppressing oxidative stress and reducing podocyte apoptosis [24]. Chan et al. reported that standard treatment plus Chinese herbal granules containing Rehmannia-6 (TCM formulation) stabilized eGFR levels in T2DM adult patients with low eGFR and UACR [25]. Gao et al. reported that the Qingxin Lianzi Yin formula improved renal histopathological damage and function in the db/db mice by influencing energy and amino acid metabolism pathways [26]. Qiyan et al. found that the JinChan YiShen TongLuo formula significantly improved renal function levels in DN rats by stabilizing HIF-1*α* levels and activating mitochondrial autophagy via PINK1/Parkin pathway [27]. Wang et al. discovered that the Da-Chai-Hu decoction enhanced renal function and relieved DN in mice by regulating glucose/lipid metabolism via suppression of the AGEs/RAGE/AKT pathway [28]. Zhang et al. found that the Dang-Gui-Bu-Xue decoction exerted therapeutic effects through dual modulation of glycation pathways and oxidative stress reduction in DN mice [29].

In previous studies, we demonstrated that TangNaikang (TNK) regulated glucose metabolism in the diabetic mice by improving the insulin sensitivity index, alleviating insulin resistance, promoting the repair and regeneration of pancreatic islet cells, and regulating abnormal lipid metabolism [30–36]. Furthermore, TNK regulated metabolic disturbances in the KKAy mice by reducing urinary protein, inhibiting FN mRNA deposition, alleviating renal tubulointerstitial fibrosis, and improving early kidney lesions [37–39]. However, the mechanism by which TNK acts on podocyte injury is unclear. As a result, this study used network pharmacology to analyze how TNK eased podocyte injury in DN, as well as investigations with in vivo db/db mice and in vitro high glucose-induced MPC5 cell models.

## 2. Materials and Methods

### 2.1. TNK Preparation

The TCM formulation TNK is composed of ginseng, *Prunella vulgaris*, *Ligustrum lucidum*, guava leaf, and *Saururus chinensis* in a 1:4:2:4:2 ratio. Ginseng, *Ligustrum lucidum*, *Prunella vulgaris*, and *Saururus chinensis* were purchased from the Beijing Shuangqiao Yanjing Pharmaceutical Company (Beijing, China). These materials met quality specifications outlined in the Chinese Pharmacopoeia 2020. The guava leaves were purchased from the Chunzhengtang Pharmaceutical Factory (Guangxi Zhuang Autonomous Region, China). It was assessed to meet the local medicinal material standards of the Guangxi Zhuang Autonomous Region. A crude mixture formulated by blending *Prunella vulgaris*, *Ligustrum lucidum*, guava leaf, and *Saururus chinensis* was extracted twice with 8 volumes of 75% ethanol and filtered. Both filtrates were mixed. Then, the ethanol was recovered to generate a clear extract with a relative density of 1.10–1.15 under conditions of 60°C–70°C, which was set aside for later use. The dregs (leftover residue) were decocted with water for 1 h, filtered, and concentrated to obtain a clear extract with a relative density of 1.10–1.15 under conditions of 60°C–70°C. This extract was then mixed with the ethanol-extracted clear extract and ginseng powder. Finally, it was completely mixed and dried to obtain the final product.

### 2.2. Ultraperformance Liquid Chromatography–Quadrupole Time-of-Flight Mass Spectrometry (UPLC-Q-TOF/MS) Analysis of TNK and TNK-Containing Serum

We weighed 0.5 g of the TNK formula sample (passed through No. 4 sieve) and placed it in a conical flask with a stopper. Then, we added 10 mL of 50% methanol solution, sealed the conical flask, and extracted by ultrasonication (100 W, 40 kHz) for 30 min. After cooling down the temperature, we added methanol to recover the loss of weight and mixed evenly. Finally, we filtered the extract and analyzed the filtrate using UPLC-Q-TOF/MS. Data were collected by Analyst TF 1.7.1 software and processed using PeakView 1.2 software. To identify the components, the mass spectrum data were initially compared to the Natural Products High-Resolution MS/MS Spectral Library database. The components were initially screened based on the score information for each chromatographic peak. Further identification of components was based on relevant literature according to the retention time of the primary mass spectrum, mass-to-charge ratio, information regarding adduct ions, isotopic information, secondary mass spectrum fragment information, and mass spectrometric fragmentation pattern of the components.

We vortexed 200 *μ*L serum with 600 *μ*L methanol for 1 min. Then, the combination was centrifuged at 12,000 rpm for 15 min. The supernatant was collected, concentrated, centrifuged, and kept at −20°C. The residue was redissolved with 200 *μ*L of 50% methanol, vortexed for 2 min, and then centrifuged at 12,000 rpm for 15 min, and the supernatant was collected. Then, it was analyzed using the UPLC-Q-TOF/MS method. Data was collected and processed using Analyst TF 1.7.1 and PeakView 1.2 software, respectively. Initially, the mass spectral data were matched with the Natural Products High-Resolution MS/MS Spectral Library database. The mass spectra of the administered biological serum samples were compared with the blank biological samples. Then, the original formula analysis results were matched with the administered serum sample data. Subsequently, we compared the primary mass retention times, mass-to-charge ratios, adduct ion information, isotope information, secondary collision energies, secondary fragment information, and literature references to identify the metabolites.

### 2.3. Network Pharmacology of TNK in DN

The chemical components' structural information, including molecular formula, molecular weight, molecular descriptor, and 2D structure file, was obtained using the TNK square mass spectrometry study and stored in the PubChem database (https://pubchem.ncbi.nlm.nih.gov/). The online docking simulation tool SuperPred (https://prediction.charite.de/) was used to predict the target of action for the TNK chemical components, and the screening condition was set to probability ≥ 0.7. The search term “diabetic nephropathy” was used to identify disease-related proteins in the OMIM (https://omim.org/), DisGeNET (https://www.disgenet.org/), and GeneCards (https://www.genecards.org/) databases. Common targets between these groups were subsequently employed to pinpoint TNK's potential therapeutic targets for DN management. Formula-target networks were constructed using Cytoscape 3.9.1, while protein interaction networks were generated via STRING database (https://string-db.org/) with a confidence threshold ≥ 0.9, featuring protein nodes connected by interaction edges. Biological functions of the targets were analyzed. The node degree (*k*) of each target in the PPI network was calculated. The average node degree (⁣^−^*k*) of the targets was used as a threshold to screen important targets. GO functional annotation (FDR < 0.05, *n* ≥ 3) of key targets was utilized using the DAVID database (https://david.ncifcrf.gov/) in the PPI network. The relevant KEGG pathways of important targets were obtained using the STRING database.

### 2.4. Animals

Cavens Laboratory Animal Company (Jiangsu, China) provided 48 C57BLKS/J-LepR^db^/LepR^db^ (db/db) mice and 12 C57BLKS/J-LepR^db/+^ (db/m) mice, all male and 11 weeks old. All mice were housed in the SPF-grade animal room of the Beijing University of Chinese Medicine (Approval No. BUCM-4-2022062303-2136) using a conventional 12-h light and 12-h dark cycle. The room temperature was kept around 24°C ± 2°C, with an air humidity of roughly 40%. They were provided with regular feed and allowed to drink water freely. They underwent a 1-week acclimation period. DN model validation required non–fasting blood glucose measurements exceeding 13.9 mmol/L in db/db mice. Mice were randomly allocated to the model group, the positive control group (metformin, 0.25 g/kg/day), the low-dose TNK group (1.14 g/kg/day), and the high-dose TNK group (4.56 g/kg/day). The db/m^+^ mice served as the normal group. In the TNK treatment groups, it was dissolved in distilled water and thoroughly stirred before gavage once a day for 8 weeks. Mice in the normal and model groups were given equal quantities of ultrapure water. Biometric monitoring included fortnightly body weight measurements and fasting blood glucose assessments following 6-h fasting periods every 2 weeks. Once every 4 weeks, mice's 24-h urine was collected for urinary protein analysis. Mice were given isoflurane anesthesia and sacrificed at the end of the treatment.

Thirty Sprague Dawley rats (200–250 g) were administered a gavage of 2.28 g/kg/day for a duration of 7 days and were kept at the SPF-grade animal room at Beijing University of Chinese Medicine (Approval No. BUCM-2023081801-3052), using a conventional 12-h light and 12-h dark cycle. The feeding environment maintains a room temperature of approximately 24°C ± 2°C and an air humidity level around 40%. Two hours after postfinal dosing, animals underwent anesthesia through intraperitoneal administration of pentobarbital sodium (45 mg/kg). Following the separation of the abdominal aorta, blood was collected using negative pressure vacuum techniques. Serum fractions were separated through cold centrifugation (4°C, 3000 rpm, 15 min) and cryopreserved at −20°C for subsequent analysis.

### 2.5. Tissue Harvest and Histological Observation

After completing an 8-week experimental protocol, mice were humanely sacrificed for biological sample collection. Blood specimens were immediately processed by centrifugation (3000 rpm, 15 min, 4°C) followed by cryopreservation at −20°C for subsequent analysis. Renal tissues were dissected and snap-frozen in liquid nitrogen prior to long-term storage at −80°C. Histopathological evaluation included hematoxylin–eosin (H&E) and periodic acid-Schiff (PAS) staining protocols, complemented by ultrastructural analysis through electron microscopy to document renal architectural alterations.

### 2.6. Biochemical Examinations

Serum total cholesterol (TC), triglyceride (TG), high-density lipoprotein (HDL), low-density lipoprotein (LDL), blood urea nitrogen (BUN), and serum creatinine (Scr) levels were measured using an automatic biochemical analyzer (BS-420, Mindray, Xiamen Haifei Biotechnology Company, China). The concentrations of 24-h urinary total albumin, serum advanced glycation end-products (AGEs), and *β*2-microglobulin (*β*2-MG) were measured using reagent kits (BC8141 [Solarbio], ZK-4500, and ZK-4977 [Zhenke Biology]), respectively.

### 2.7. Quantitative Real-Time PCR (RT-qPCR)

Homogenize mice kidney tissue, and isolate total RNA strictly adhering to basic experimental requirements. The tissue homogenate is subsequently combined with a lysis buffer, after which centrifugation is performed to obtain the supernatant. The supernatant is then subjected to a genomic DNA removal column, followed by centrifugation, and the filtrate is collected. Ethanol is incorporated into the retained filtrate, mixed thoroughly, and subsequently transferred to an RNase-free adsorption column CR4, after which centrifugation is performed. RNase-free double-distilled water is ultimately dispensed into the adsorption column, and following centrifugation, the RNA solution is acquired. The HiScript III RT SuperMix for qPCR (Cat. R323-01, Vazyme) is utilized for reverse transcription to obtain the cDNA solution. The experiment utilizes the BrightCycle Universal SYBR Green qPCR Mix with UDG (Cat. RK21219, ABclonal Biotech) with the Applied Biosystems 7500 qPCR System (Thermo Fisher Scientific). *β*-Actin served as the normalization, with relative expression levels calculated through the 2^−*ΔΔ*CT^ method. The primers are listed in Supporting Information 1: Table S1.

### 2.8. Multiplex Immunofluorescence Staining

We dewaxed 4–5 *μ*m thick kidney tissue sections, performed antigen retrieval, and washed with TBST. The activity of endogenous peroxidase in the sections was inhibited. Then, following blocking procedures, tissue sections were subjected to overnight incubation at 4°C with specific primary antibodies: PI3K (Cat. 4292S, Cell Signaling Technology), AKT (Cat. 4691S, Cell Signaling Technology), SYNPO (Cat. 21064-1-AP, Proteintech), nephrin (Cat. ab216341, Cell Signaling Technology), CD2AP (Cat. #2135, Cell Signaling Technology), SHIP2 (Cat. ab166916, Abcam), podocin (Cat. ab181143, Abcam), IRS2 (Cat. 20702-1-AP, Proteintech), and GLUT4 (Cat. 66846-1-Ig, Proteintech). Subsequently, the sections were treated with HRP-conjugated secondary antibodies (GB23303, dilution 1:600, ServiceBio) and fluorescent TSA dyes (iF440, iF488, iF546, iF594, and iF700) sequentially. The nuclei were counterstained with DAPI, and autofluorescence was quenched. Finally, the sections were analyzed, and the percentages of positive cells in the glomerulus were quantified using the QuPath software (Version 0.6.0).

### 2.9. Cell Culturing and Cell Viability Assay

MPC5 cells (FH1009, Fuheng Biology, Shanghai, China) were grown in the DMEM complete medium (FH-mpc-5, Fuheng Biology, Shanghai, China) until 80% confluence. Subsequently, the cells were digested and counted. Then, they were cultured in low-glucose DMEM complete medium (5.5 mmol/L) for four or more passages. The logarithmic growth phase's cells exhibited stable differentiation and maturation.

The appropriate serum concentration for delivering TNK was established according to the CCK-8 assay instructions. MPC5 cells were subsequently categorized into four distinct groups: normal group (5.5 mmol/L glucose), model group (25 mmol/L glucose), low-dose TNK-containing serum group (25 mmol/L glucose + 5% TNK-containing serum, TNK-L), and high-dose TNK-containing serum group (25 mmol/L glucose + 20% TNK-containing serum, TNK-H). Subsequently, the proliferation of these groups was assessed after 24 h using the CCK-8 assay. In a 96-well plate, we seeded 1 × 10^4^ cells per 200 *μ*L/well with low serum DMEM medium (2% FBS).

Cells were maintained at 37°C with 5% CO_2_ for 18–24 h prior to medium replacement. Subsequently, cells were cultured in serum-free, low-glucose DMEM medium for 12 h to synchronize them at the G_0_ phase. After observing the cellular status under the microscope, the medium was changed. With the exception of the normal group, all other groups are stimulated with high glucose (25 mmol/L glucose). TNK groups were cultured in TNK-containing serum following high glucose induction. After 24 h of incubation, the medium was replaced with fresh medium containing 10% CCK-8 assay. Cells were incubated in the dark for 40 min. Each group was set up with six replicative wells. The experiment was repeated three times. The absorbance of each well was detected at 450 nm by using a microplate reader.

After 24 h of intervention with the TNK-containing serum, the multiple immunofluorescence method was also employed to identify changes in protein levels of normal, high glucose-induced MPC5 cells, LY294002 (3 *μ*L/L, Cat. GC15485, GLPBIO), TNK-L, TNK-H, TNK-L with LY294002, and TNK-H with LY294002 groups following the steps outlined above. MPC5 cells were digested, counted, and seeded onto sterile coverslips in 6-well plates at a density of 1.5 × 10^6^ cells/mL. Then, the cells were fixed with 4% paraformaldehyde, rinsed with PBS, and blocked. Subsequently, multiple markers were analyzed simultaneously on each slide by labeling with antibodies against SHIP2, CD2AP, podocin, SYNPO, nephrin, P-PI3K (Cat. 4292S, Cell Signaling Technology), P-AKT (Cat. 4060, Cell Signaling Technology), PI3K, and AKT. The cell slides were then individually coated with HRP-conjugated secondary antibody (GB23303, ServiceBio) and fluorescent TSA dyes (iF440, iF488, iF546, iF594, and iF700), respectively. The nuclei underwent counterstaining with DAPI, and autofluorescence was quenched. Images were subsequently captured by laser confocal microscope. A quantitative analysis of fluorescence intensity was performed utilizing QuPath software (Version 0.6.0).

### 2.10. Western Blot Analysis

Western blotting was employed to ascertain the expression levels of P-PI3K, PI3K, P-AKT, AKT, SYNPO, nephrin, and SHIP2 proteins, in perfect compliance with fundamental experimental protocols. The following reagents were utilized: Total Protein Extraction Kit (Cat. BC3710, Solarbio), *β*-actin antibody (Cat. 4970, CST), and goat anti-rabbit IgG secondary antibody (Cat. SA00001-2, Proteintech). The final results were presented as relative expression levels normalized to *β*-actin, utilizing Image Lab Software (Version 6.1, Bio-Rad).

### 2.11. Statistical Analysis

Quantitative data are expressed as mean values ± standard deviation. Statistical evaluations were performed utilizing IBM SPSS software (Version 23.0, United States), while graphs were created through GraphPad Prism (Version 9.0, United States). For datasets demonstrating normal distribution, one-way analysis of variance (ANOVA) was employed, with post hoc analyses conducted using either Fisher's LSD method or Dunnett's multiple comparison test. Nonparametric datasets underwent assessment through the Kruskal–Wallis test. Statistical significance thresholds were established at *p* value < 0.05.

## 3. Results

### 3.1. UPLC-Q-TOF/MS and Network Pharmacology Analysis of TNK and TNK-Containing Serum

**Figure 1:**
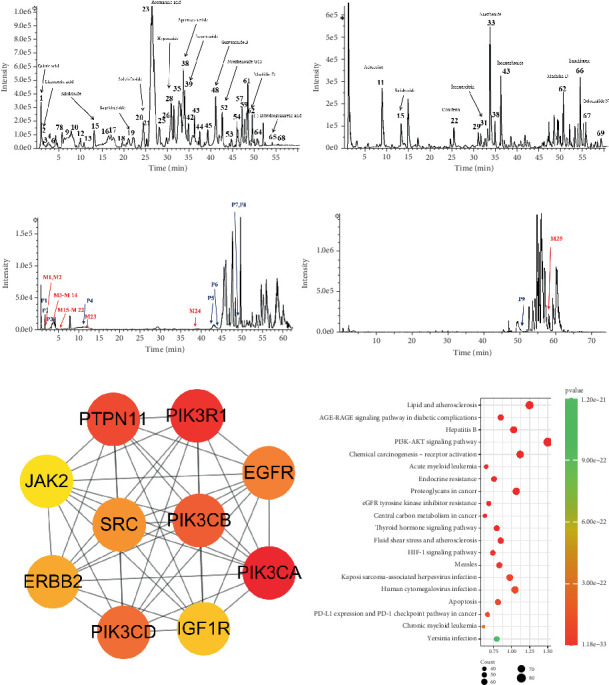
Mass spectrometry spectrum of TNK and TNK-containing serum and network pharmacology results for the mechanisms of TNK. (a) Base peak chromatogram (BPC) of TNK in UPLC-HRMS—negative ion mode and positive ion mode. (b) Base peak chromatogram (BPC) of TNK-containing serum in UPLC-HRMS—negative ion mode and positive ion mode. (c) PPI analysis and KEGG enrichment analysis of potential therapeutic targets upon systemic circulation of TNK.

We performed UPLC-Q-TOF/MS analysis of the TNK formula and identified 69 compounds, including 12 from ginseng, 12 from *Saururus chinensis*, 24 from *Ligustrum lucidum*, 24 from *Prunella vulgaris*, and 13 from *Psidium guajava* leaves, based on the classification of medicinal materials. The identified compounds included flavonoids, flavonoid glycosides, iridoid glycosides, triterpenoids, triterpenoid saponins, lignans, phenylethanoid glycosides, and unsaturated fatty acids. Components of UPLC-Q-TOFMS analysis of TNK are listed in Supporting Information 2: Table S2. The mass spectrometry spectrum and both base peak chromatograms (BPCs) of TNK in UPLC-HRMS, both in negative and positive ion modes, are shown in Figure 1.

UPLC-Q-TOF/MS analysis of the TNK-containing serum identified 34 compounds including 9 TNK prototype components and 25 metabolites, including limonexic acid, gallic acid, salidroside, pinostrobin, sauchinone, and protopanaxatriol. Components of UPLC-Q-TOFMS analysis of TNK-containing serum are listed in Supporting Information 3: Table S3. The mass spectrometry spectrum and both BPCs of TNK-containing serum in UPLC-HRMS, both in negative and positive ion modes, are shown in Figure 1.

We obtained a total of 4948 DN-associated targets from the OMIM, DisGeNET, and GeneCards databases and identified 1033 drug-related targets via SuperPred. The findings of the Venn analysis indicated the existence of 553 potential interaction targets between drug components and DN. PPI network analysis demonstrated that PIK3R1 and PIK3CA were central targets of TNK. Subsequently, a KEGG enrichment analysis was conducted on these 553 potential interaction targets. RAGE and PI3K were the main target pathways of TNK according to the KEGG enrichment analysis, indicating that the PI3K/AKT pathway was substantially related to TNK. Network pharmacology results for the mechanisms of TNK in DN are shown in Figure 1.

### 3.2. TNK Improves Metabolism Disorders and Renal Function and Structure in the db/db Mice

**Figure 2:**
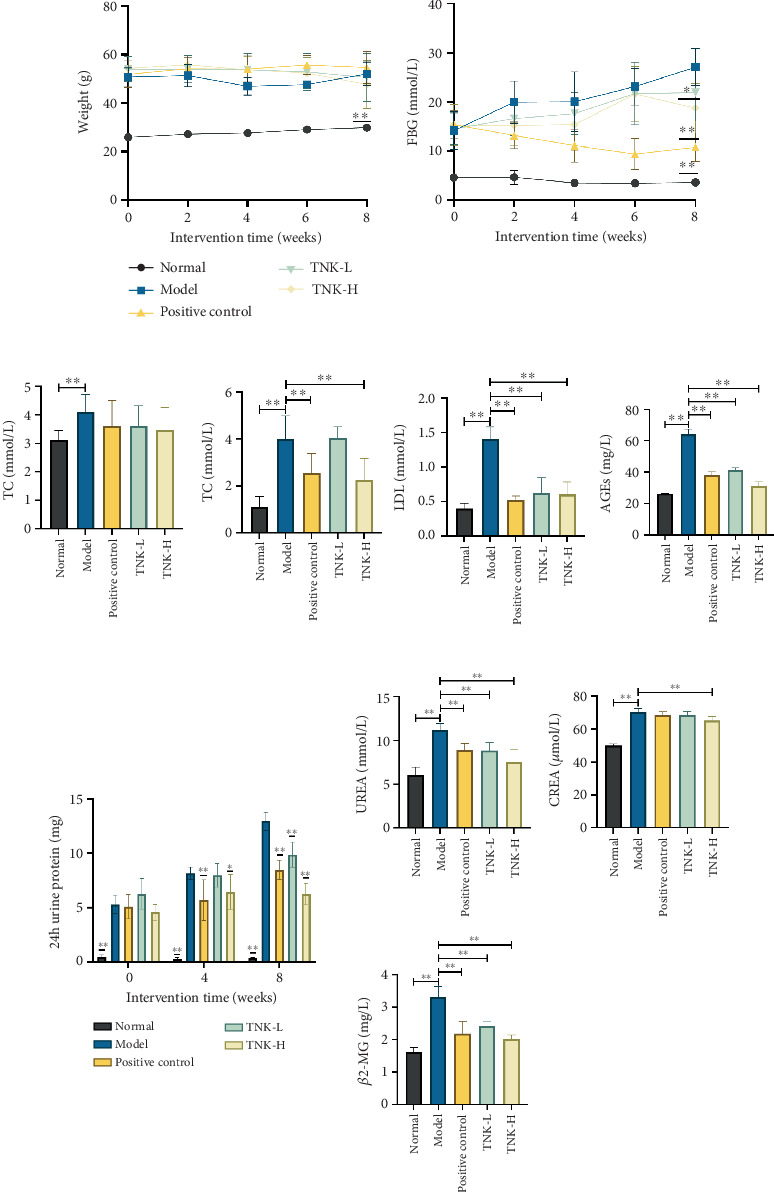
Effects of TNK on metabolism disorders and renal function in db/db mice. (a) Body weight and fasting blood glucose levels of mice during intervention time. (b) The effects of TNK on serum levels of TC, TG, HDL, LDL, and AGEs in mice, respectively. (c) The effects of TNK on urine protein and UREA, CREA, and *β*2-MG of mice during intervention time. Normal: db/m + mice treated with vehicle. Model: db/db mice treated with vehicle. Positive control: db/db mice treated with 0.25 g/kg/day metformin. TNK-L: db/db mice treated with 1.14 g/kg/day TangNaikang. TNK-H: db/db mice treated with 4.56 g/kg/day TangNaikang. One-way ANOVA analysis applied for statistical analysis, *N* = 6–8/group. ⁣^∗^*p* < 0.05;^∗∗^*p* < 0.01.

The serum test findings of db/db mice indicated a reduction in the expression levels of AGEs in the positive control and TNK groups, implying an enhancement in glucose metabolism, with significant reductions compared to the model group (*p* < 0.01). Lipid profile analysis revealed substantially elevated TC, TG, and LDL concentrations in the model group relative to normal controls (*p* < 0.01). The positive control and TNK groups exhibited significantly lower LDL levels compared to the model group (*p* < 0.01). Additionally, the positive control and TNK groups exhibited elevated HDL levels compared to the model group, with no significance. Effects of TNK on metabolism disorders in db/db mice are shown in Figure 2.

Compared to the normal group, the model group's 24-h urine protein levels gradually increased over the intervention (*p* < 0.01). However, after 8 weeks of intervention, the positive control and TNK groups had significantly lower 24-h urine protein levels than the model group (*p* < 0.01). The model group had considerably higher serum levels of BUN, Scr, and *β*2-MG than the normal group, indicating severe renal dysfunction (*p* < 0.01). In comparison with the model group, the positive control and TNK groups showed significantly lower levels of BUN (*p* < 0.01). Furthermore, compared to the model group, the *β*2-MG index, which reflects glomerular filtration function, had a significant reduction in the positive control and TNK groups (*p* < 0.01). Effects of TNK on renal function in db/db mice are shown in Figure 2.

**Figure 3:**
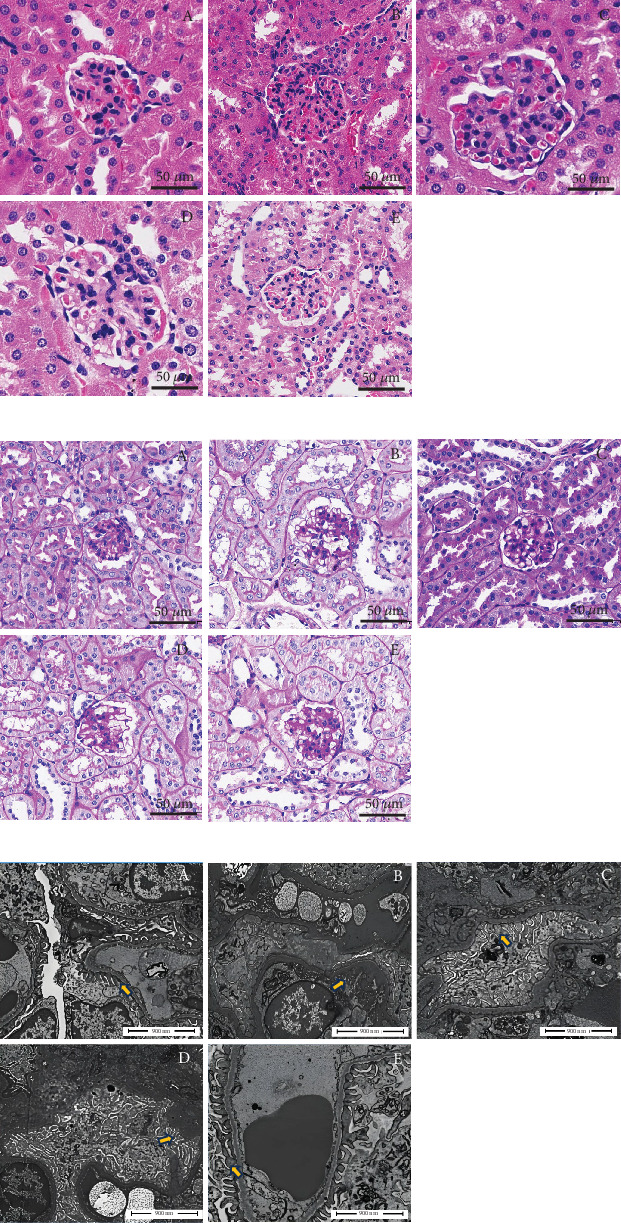
Effects of TNK on renal structure in db/db mice. Effects of TNK on pathological structure (a) H&E, (b) PAS staining, and (c) ultrastructure in mice. (A) Normal: db/m+ mice treated with vehicle. (B) Model: db/db mice treated with vehicle. (C) Positive control: db/db mice treated with 0.25g/kg/day metformin. (D) TNK-L: db/db mice treated with 1.14g/kg/day TangNaikang. (E) TNK-H: db/db mice treated with 4.56 g/kg/day TangNaikang.

Based on the H&E and PAS staining results, the kidneys in the normal group showed regular and complete glomerular structure, smooth basement membrane, neatly formed renal tubules, and no invasion of inflammatory cells in the renal interstitium. The kidney sections in the model group showed increased glomerular volume, basement membrane thickening, mesangium expansion, renal tubule enlargement, disordered structure, renal interstitial edema, and inflammatory cell infiltration. Compared with the model group, the TNK-H group had a much smaller glomerular volume, a more complete structure, less basement membrane thickening, absence of mesangial expansion, low interstitial edema, and less inflammatory cell infiltration (Figure 3). The scanning electron microscope findings indicated that the podocyte foot processes in the normal group exhibited normal and distinct structures representing a palisading pattern and exhibited tight adherence to the external surface of the capillary basement membrane. In the model group, the podocyte foot processes disappeared and fused in segments. Compared to the model group, the TNK-H group had a considerable reduction in segmental disappearance and fusion of the podocyte foot processes. Effects of TNK on renal structure in db/db mice are shown in Figure 3.

### 3.3. TNK Alleviates Podocyte Injury by Activating PI3K/AKT Signaling Pathway

**Figure 4:**
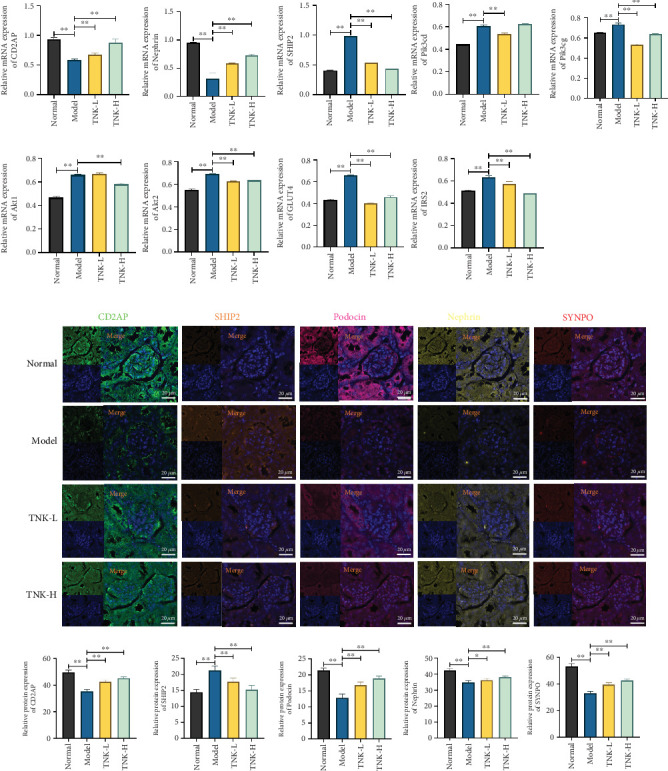
Effects of TNK on mRNA and protein expression levels of the PI3K/AKT signaling pathway in mice. (a) Relative mRNA expression levels of CD2AP, Nephrin, SHIP2, Pik3cd, Pik3cg, Akt1, Akt2, IRS2, and GLUT4 in mice, respectively (N = 3). (b) Effects of TNK on CD2AP, SHIP2, Podocin, Nephrin, SYNPO, PI3K, AKT, GLUT4, and IRS2 protein expression levels with multiplex immunohistochemical in mice, respectively (N = 3). Normal: db/m+ mice treated with vehicle. Model: db/db mice treated with vehicle. TNK-L: db/db mice treated with 1.14g/kg/day TangNaikang. TNK-H: db/db mice treated with 4.56 g/kg/day TangNaikang. One-way ANOVA analysis applied for statistical analysis. ⁣^∗^*p* < 0.05 and ^∗∗^*p* < 0.01.

RT-qPCR results demonstrated that *CD2AP* and *nephrin* mRNA expression levels were considerably lower in the model group, while *SHIP2* mRNA expression levels were higher compared to the normal group (*p* < 0.01). *CD2AP* and *nephrin* mRNA expression levels were significantly higher in the TNK groups compared with the model group (*p* < 0.01). Conversely, the *SHIP2* mRNA expression levels were significantly lower in the TNK groups compared to the model group (*p* < 0.01). The TNK-L group showed significantly lower *Pik3cd* mRNA expression levels than the model group (*p* < 0.01). The TNK-L and TNK-H groups had significantly lower *Pik3cg* mRNA expression levels than the model group (*p* < 0.01). The TNK-H group showed significantly lower *Akt1* mRNA expression levels than the model group (*p* < 0.01). TNK groups had significantly lower *Akt2* and *IRS2* mRNA expression levels than the model group (*p* < 0.01). Furthermore, the TNK groups had significantly lower *GLUT4* mRNA expression levels than the model group (*p* < 0.01). Effects of TNK on mRNA expression levels of the PI3K/AKT signaling pathway in mice are shown in Figure 4.

**Figure 5:**
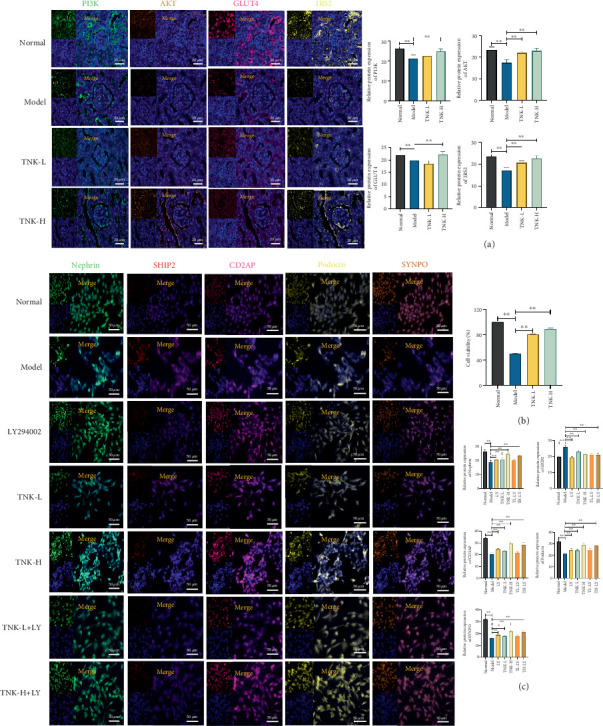
(a) The effects of TNK on the protein expression levels of PI3K, AKT, GLUT4, and IRS2 were assessed using (a) multiplex immunohistochemistry in mice, with animal grouping illustrated in Figure 4. (b) Cell viability of MPC5 cells in all groups. (c) Effects of TNK-containing serum on the protein expression levels of Nephrin, SHIP2, CD2AP, Podocin, and SYNPO with multiplex immunohistochemical in MPC5 cells. Normal group: glucose 5.5 mmol/L. Model group: high-glucose (25 mmol/L). LY294002 group: blocker of PI3K/AKT pathway, 3 μmol/L. TNK-L: TNK-containing low-dose serum group (glucose 25 mmol/L + 5% TNK-containing serum, TNK-L). TNK-H: TNK-containing high-dose serum group (glucose 25 mmol/L + 20% TNK-containing serum, TNK-H). TNK-L+LY: TNK-containing low-dose serum with LY294002 group (glucose 25 mmol/L + 5% TNK-containing serum, TL+LY). TNK-H+LY: TNK-containing high-dose serum with LY294002 group (glucose 25 mmol/L + 20% TNK-containing serum, TH+LY). One-way ANOVA analysis applied for statistical analysis, N = 3/group. ⁣^∗^*p* < 0.05 and ^∗∗^*p* < 0.01.

The model group showed considerably greater SHIP2 expression in the glomeruli than the normal group, according to multiple immunofluorescence results. However, CD2AP, podocin, SYNPO, and nephrin protein expression dramatically decreased (*p* < 0.01). The TNK groups showed substantially reduced SHIP2 expression levels in the glomeruli compared to the model group (*p* < 0.01). The model group showed substantially decreased PI3K, AKT, IRS2, and GLUT4 expression levels compared to the normal group (*p* < 0.01). The TNK-H group showed significantly greater PI3K, AKT, IRS2, and GLUT4 protein expression levels than the model group (*p* < 0.01). Effects of TNK on protein expression levels of the PI3K/AKT signaling pathway in mice are shown in Figures 4 and 5.

### 3.4. The Effects of TNK-Containing Serum on MPC5 Cell Injury and PI3K/AKT Signaling Pathway

As shown in Figure 5, the TNK-containing serum significantly increased the viability of MPC5 cells after 24 h compared to the MPC5 cells of the model group (*p* < 0.01). Multiplex immunofluorescence experiments showed that SHIP2 expression was significantly higher in the MPC5 cells of the model group compared to the MPC5 cells of the normal group. Conversely, CD2AP, podocin, SYNPO, and nephrin expression levels were significantly lower in the MPC5 cells of the model group compared to the MPC5 cells of the normal group (*p* < 0.01). The MPC5 cells of the TNK-H-LY group had significantly decreased SHIP2 expression levels, while had considerably greater levels of CD2AP, podocin, SYNPO, and nephrin than the MPC5 cells of the model group (*p* < 0.01).

**Figure 6:**
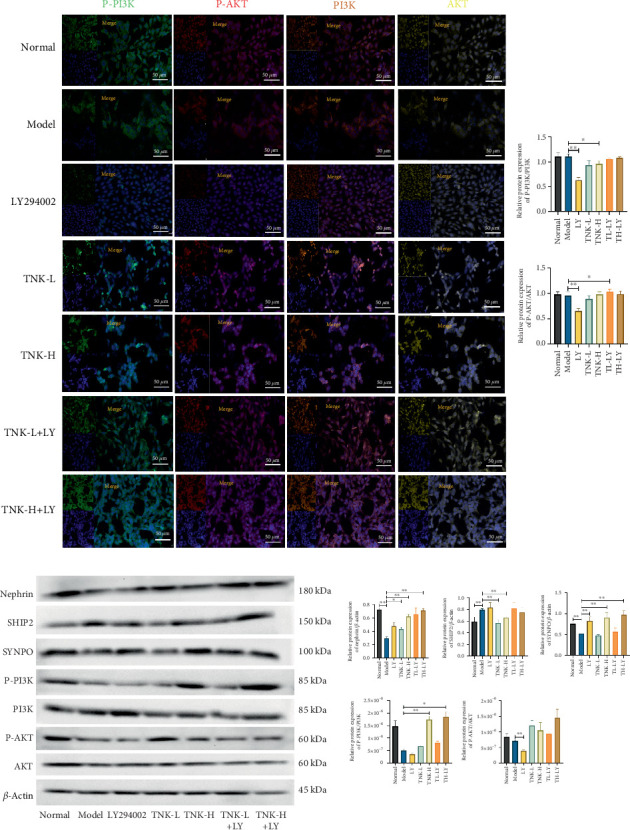
Effects of TNK-containing serum on MPC5 cells by influencing nephrin, SHIP2, and SYNPO protein expression and PI3K/AKT pathway. (a) P-PI3K, P-AKT, PI3K, and AKT protein expression with multiplex immunohistochemical in MPC5 cells. (b) AKT, P-AKT, PI3K, P-PI3K, SYNPO, SHIP2, and nephrin protein expression with western blotting in MPC5 cells, with cell grouping illustrated in Figure 5. One-way ANOVA analysis applied for statistical analysis, *N* = 3/group. ⁣^∗^*p* < 0.05;^∗∗^*p* < 0.01.

Western blotting analysis results also showed that the MPC5 cells of the model group had considerably increased expression of SHIP2 and decreased expression of SYNPO and nephrin proteins compared to the MPC5 cells of the normal group (*p* < 0.01). However, compared to the model group, MPC5 cells of the TNK-H group revealed considerably decreased SHIP2 expression (*p* < 0.01) and significantly greater levels of SYNPO and nephrin (*p* < 0.01). Moreover, the MPC5 cells of the TNK-H group showed higher expression of P-PI3K compared to the MPC5 cells of the model group (*p* < 0.01). As shown in Figures 5 and 6, the protective effects of TNK-containing serum on MPC5 cells are demonstrated by influencing nephrin, SHIP2, and SYNPO protein expression, as well as the PI3K/AKT pathway.

## 4. Discussion

Clinical and experimental studies have demonstrated that aberrant insulin signaling contributes significantly to podocyte injury and DN development [40]. The insulin signaling pathway regulates various responses of podocytes. Insulin binds to the insulin receptor and phosphorylates insulin receptor substrates (IRSs), such as IRS1. IRSs are adaptor proteins that bind to PI3K or growth factor receptor-bound protein and activate downstream targets such as AKT, GSK3, eNOS, Ras, ERK, and PKC. This causes a variety of consequences in podocytes, including changes in mitochondrial activity, autophagy, ER stress, VEGF-A production, actin dynamics, albumin permeability, and calcium flow [41–43]. Welsh et al. found that specific podocyte insulin receptor deletion mice had DN-like glomerular lesions, proteinuria, and glomerulosclerosis without higher blood glucose levels [44, 45]. Aberrant insulin signaling causes dramatic alterations in podocyte phenotype and death, showing that the insulin signaling pathway is critical for maintaining the glomerular filtration barrier integrity [46]. SHIP2 is a lipid phosphatase that can hydrolyze PIP3 into PIP2, thereby inhibiting downstream signaling pathways activated by PI3K [21, 47]. Paulina et al. reported that inhibiting SHIP2 activity improved oxidative stress in the podocytes lacking CD2AP [48]. Polianskyte-Prause et al. reported that metformin ameliorated reduced glucose uptake in the SHIP2-overexpressing myotubes by slowing down GLUT4 endocytosis and suppressed podocyte apoptosis by inhibiting SHIP2 overexpression [49]. In studies, sulfonyl aniline analogs were tested as SHIP2 inhibitors in the rat L6 myoblast model, revealing that these inhibitors can induce AKT activation and enhance GLUT4 expression on the plasma membrane. This finding further underscores SHIP2's negative regulatory role in insulin regulation from another perspective [50]. Consequently, SHIP2 plays a crucial part in inhibiting podocyte insulin sensitivity.

TNK is a clinical empirical formula for treating diabetes and its complications under the guidance of TCM theory. TNK is composed of *Prunella vulgaris*, guava leaf, ginseng, *Ligustrum lucidum*, and *Saururus chinensis* and is registered under a National Patent Number ZL02153751.8. Its benefits include clearing heat, resolving bulk, and nourishing qi and yin. Our data demonstrated that the TNK preparation consisted of 69 chemical components, including 12 from *Panax ginseng*, 12 from *Saururus chinensis*, 24 from *Ligustrum lucidum*, 24 from *Prunella vulgaris*, and 13 from *Psidium guajava* leaves, as classified by medicinal materials. The formulation included flavonoids, flavonoid glycosides, iridoid glycosides, triterpenoids, triterpenoid saponins, lignans, phenylethanoid glycosides, and unsaturated fatty acids. After administration of TNK, we identified 34 components in the murine serum samples, including 9 drug prototype components and 25 metabolites. This included danshensu, salidroside, and quercetin. Therefore, we speculated that TNK exerted its pharmacological effects through these active components.

In DN rats, Karunasagara et al. found that ginseng could alleviate renal inflammation and fibrosis by reducing the production of TGF-*β*1, KIM1, and AGE proteins [51]. Park et al. found that ginsenoside Rb1 can inhibit fibrosis in primary mesangial cells induced by high glucose by decreasing the phosphorylation of p38 MAPK, JNK/SAPK, and AKT proteins [52]. Peng et al. found that ursolic acid in *Prunella vulgaris* can alleviate inflammation and oxidative stress in rats with acute kidney injury by inhibiting the activities of STAT3, NF-*κ*B, and caspase-3 [53]. Tang et al. reported that quercetin enhanced glucose metabolism, improved renal histopathological alterations, and reduced levels of AGEs in streptozotocin-induced DN rats [54]. Liu et al. demonstrated that quercetin could mitigate podocyte apoptosis in db/db mice and high glucose-induced podocytes by regulating the expression of Bax and Bcl-2 proteins [55]. Feng et al. demonstrated through in vivo and in vitro experiments that quercetin administration mitigated iron-dependent cell death in DN by modulating iron metabolism regulators (TFR-1 reduction and GPX4/FTH-1 elevation) and activating the Nrf2/HO-1 antioxidant pathway [56].

Yang et al. revealed that *Prunella vulgaris* and its components, including quercetin, luteolin, and kaempferol, may exhibit protective effects against COVID-19-related acute kidney injury utilizing network pharmacology and bioinformatics methodologies [57]. Liu et al. investigated the impact of quercetin on DN mice subjected to a high-fat diet in combination with STZ and HK-2 cells, discovering that quercetin inhibited cell apoptosis via the PI3K/AKT signaling pathway, exerting a protective effect [58]. Li et al. found that buckwheat hull flavonoids decreased AGEs and improved the renal function of db/db mice by modulating the AGE–RAGE pathway [59]. Diao et al. administered Huangkui capsule to db/db mice, a clinical prescription for DN patients in China, observing that its protective effect is associated with metabolic pathways involving dehydrogenation, deglycosylation, glucuronidation, and sulfation [60].

**Figure 7:**
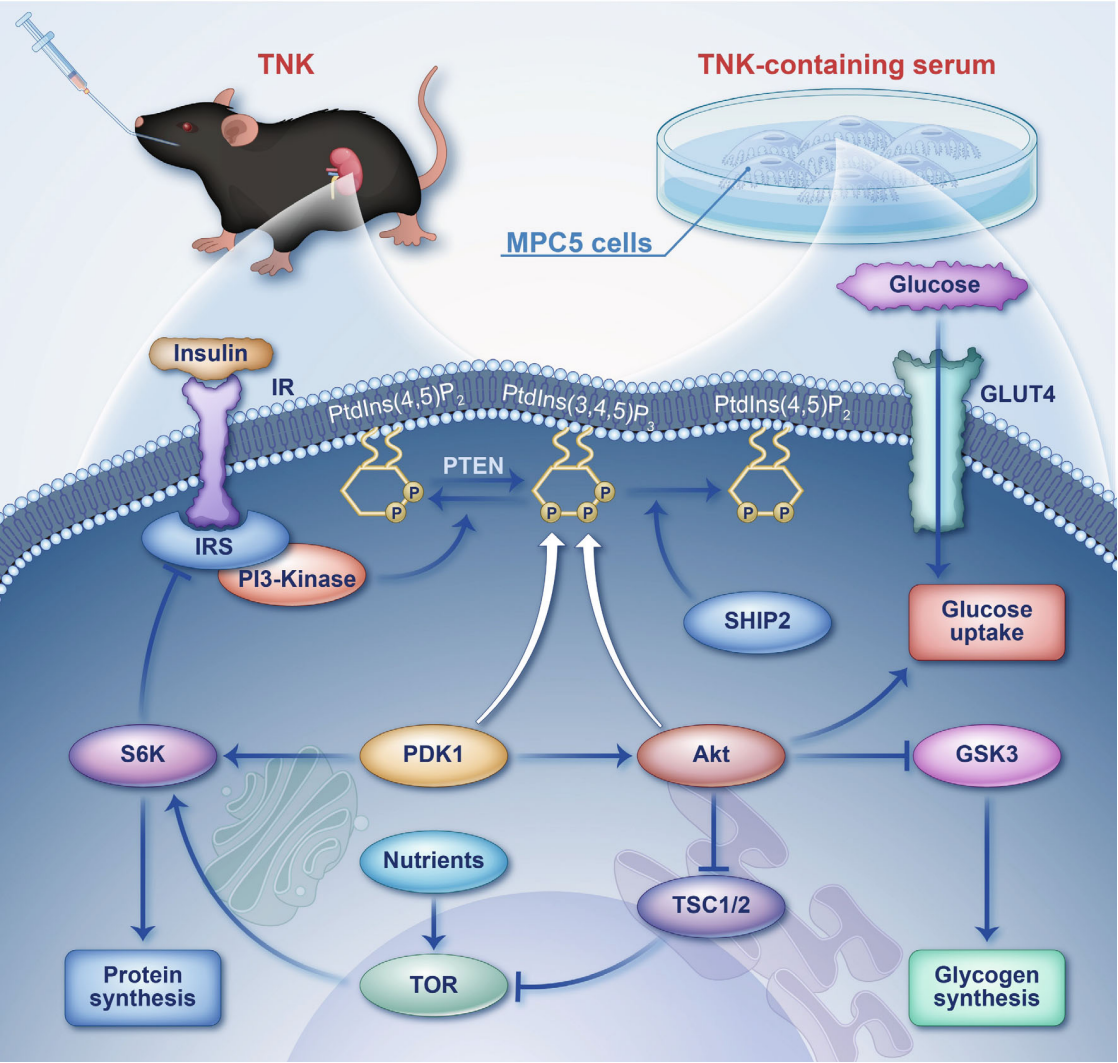
TNK intervention ameliorated DN by modulating insulin sensitivity through downregulating SHIP2, improving the PI3K/AKT signaling pathway (by Figdraw).

Our study showed that TNK improved the renal ultrastructure in the db/db mice. After analyzing the mechanism of TNK's intervention in DN through network pharmacology, we found it to be intimately associated with the PI3K/AKT pathway. Nevertheless, given that network pharmacology constitutes a dynamic database, the data tags pertaining to the compounds within TNK and the SHIP2 in DN remain incomplete. Consequently, the correlation between TNK and SHIP2 has yet to be manifested. Therefore, utilizing the aforementioned methods, we have established a link between TNK, SHIP2, and the PI3K/AKT pathway and delved into the pertinent mechanisms, as shown in Figure 7. Based on preliminary pharmacodynamics testing results, the TNK-H group performed better. Consequently, during the mechanism validation, comparisons were solely conducted among the normal group, the model group, the TNK-L group, and the TNK-H group. Furthermore, the expression levels of PI3K, AKT, IRS2, GLUT4, CD2AP, podocin, SYNPO, and nephrin proteins were significantly improved in the kidneys of the TNK-H group mice compared to the model group. In the RT-qPCR experiment, the primers targeting *Pik3ca* and *Pik3cb* isoforms failed to amplify successfully, consequently leading to the nonreporting of their respective results. Therefore, we conclude that TNK exerts its effects on the PI3K/AKT signaling pathway by downregulating SHIP2, thereby improving insulin resistance and exerting protective effects on podocyte injury in the DN mice and high glucose-stimulated MPC5 cells. However, our research still has some limitations. For example, in vivo experiments, employing SHIP2 gene knockout mice for mechanism validation, would offer a more compelling approach to ascertain the regulatory role of TNK in DN. In vitro experiments, we have not established a model of SHIP2 gene-transfected podocytes for investigation. Consequently, our findings only suggested that the protective effects of TNK-containing serum on MPC5 cells induced by high glucose is associated with the PI3K/AKT signaling pathway and SHIP2 factor, without yet constituting a complete and compelling evidence base to support this conclusion. To confirm the underlying mechanisms of action, more investigations are needed.

## 5. Conclusions

TNK formula improves db/db mice's glucose and lipid metabolism, as well as renal structure and function. It also reduces 24-h urinary protein levels, improves glomerular structure, and significantly reduces the segmental disappearance and fusion of the podocyte foot processes in the db/db mice. The results of multiple immunofluorescence showed that TNK lowers the SHIP2 expression while increasing PI3K, AKT, IRS2, GLUT4, CD2AP, podocin, SYNPO, and nephrin expression. RT-qPCR results showed that TNK reduces the mRNA expression levels of *SHIP2*, *Pik3cg*, *Akt1*, *Akt2*, *IRS2*, and *GLUT4* in the db/db mice while increasing the mRNA expression levels of *nephrin* and *CD2AP*.

The in vitro experiments showed that TNK-containing serum greatly enhanced the viability of high glucose-induced MPC5 cells by preventing apoptosis. Multiple immunofluorescence results showed that TNK-containing serum reduced the expression levels of SHIP2 protein while increasing the expression levels of CD2AP, podocin, SYNPO, and nephrin proteins in the high glucose-induced MPC5 cells. Western blotting results revealed that the TNK-containing serum reduced SHIP2 protein expression while increasing SYNPO, nephrin, P-PI3K, and P-AKT proteins in high glucose-induced MPC5 cells.

## Data Availability

All data generated or analyzed during this study are available from the corresponding authors upon reasonable request.

## Supporting Information

Supporting Information 1 Table S1: Primers used in RT-qPCR.

Supporting Information 2 Table S2: UPLC-Q-TOF/MS analysis of TangNaikang.

Supporting Information 3 Table S3: UPLC-Q-TOF/MS analysis of TangNaikang-containing serum.
