# Supplementary material for: TangNaikang Formula Alleviates Podocyte Injury in Diabetic Nephropathy by Modulating the SHIP2/PI3K/AKT Pathway
Source: J Diabetes Res. 2025 Nov 19;2025:6568591. doi: 10.1155/jdr/6568591 (PMC12657081; doi:10.1155/jdr/6568591)
Supplement: Supporting Information 1 — Table S1: Primers used in RT-qPCR. [file 6568591.f1.pdf]

Table S1 Primers used in RT-qPCR

	Forward primer	Reverse primer
β-actin	GGCTGTATTCCCCTCCATCG	CCAGTTGGTAACAATGCCATGT
IRS2	ACGTCGTCGCCACAGTTCAGA	ACATTTTCCACAGAGGCCGAAT
SHIP2	ACTCTGCGTCCTGTATCAAAAG	CAGGGCACAAACAAGACCC
GLUT4	ACACTGGTCCTAGCTGTATTCT	CCAGCCACGTTGCATTGTA
Nephrin	ATGGGAGCTAAGGAAGCCACA	CCACACCACAGCTTAACTGTC
CD2AP	AAGGAGAACTAAATGGGAGACGA	CCGTTTGATGGGCAAATTGTCA
Pik3ca	ATTTGGCTATAAGCGGGAAC	TTGCTAGGTAAGCCTTGTAACAC
Pik3cb	TTATGTCCTCGGCATTGGT	AATAAAAGGTACTCGCTCCC
Pik3cd	GGAACAGCCATTCTCCATTGAGC	CTGAGCATACATTCACCTCCGAG
Pik3cg	CCCCGAGAGCTTTAGAGTTCC	TTGGAGGCCATCACTTTGCATT
Akt1	TGCCCTGGACTACTTGCACT	ATCTTGATGTGCCCGTCCT
Akt2	CACCCTTCAAACCTCAGGTCAC	GTCCAGGCTGTCATATCGGTC
