# Supplementary material for: TangNaikang Formula Alleviates Podocyte Injury in Diabetic Nephropathy by Modulating the SHIP2/PI3K/AKT Pathway
Source: J Diabetes Res. 2025 Nov 19;2025:6568591. doi: 10.1155/jdr/6568591 (PMC12657081; doi:10.1155/jdr/6568591)
Supplement: Supporting Information 2 — Table S2: UPLC-Q-TOF/MS analysis of TangNaikang. [file 6568591.f2.pdf]

Table S2. UPLC-Q-TOFMS analysis of Tangnaikang

Serial number	Time (min)	Jiahe Ionic	*m/z* Actual value	*m/z* Theoretical value	ppm	Molecular formula	Molecular weight	Components	MS/MS data	Source
1	0.812	$[M-H]^-$	191.0559	191.0561	-1.0	$C_7H_{12}O_6$	192.06	Quinic acid	191.0552; 127.0394; 85.0290	Prunella Vulgaris
2	1.173	$[M-H]^-$	191.0193	191.0197	-2.1	$C_6H_8O_7$	192.12	Limonexic acid	111.0082; 87.0084; 85.0291; 67.0187; 57.0342	Ligustrum lucidum
3	1.787	$[M-H]^-$	169.0139	169.0142	-1.8	$C_7H_6O_5$	170.02	Gallic acid	169.0134; 125.0237; 97.0294; 79.0187; 51.0239	Prunella Vulgaris, Ligustrum lucidum
4	2.285	$[M-H]^-$	179.0347	179.035	-1.7	$C_9H_8O_4$	180.04	Caffeic acid	179.0336; 135.0446; 89.0394; 79.0550	Prunella vulgaris, Ligustrum lucidum, Saururus chinensis
5	2.403	$[M-H]^-$	197.0457	197.0455	1.0	$C_9H_{10}O_5$	198.05	Salvianic acid A	179.0339; 151.0395; 135.0446; 123.0445; 109.0287; 72.9928	Prunella Vulgaris
6	3.025	$[M-H]^-$	153.0193	153.0193	0.0	$C_7H_6O_4$	154.03	Protocatechuic acid	109.0293; 108.0214; 91.0189; 81.0362; 65.0031	Prunella vulgaris, Saururus chinensis
7	4.362	$[M-H]^-$	331.0671	331.0671	0.0	$C_{13}H_{16}O_{10}$	332.07	1-O-Galloyl-β-D-glucose	271.0443; 211.0236; 169.0135	Guava leaves
8	5.87	$[M-H]^-$	331.0663	331.0671	-2.4	$C_{13}H_{16}O_{10}$	332.07	6-O-Galloyl-β-D-glucose	271.0444; 211.0237; 169.0136	Guava leaves
9	6.991	$[M-H]^-$	167.0348	167.035	-1.2	$C_8H_8O_4$	168.04	Vanillic acid	123.0451; 108.0212; 93.0343	Prunella Vulgaris
10	8.193	$[M-H]^-$	153.0557	153.0555	1.3	$C_8H_{10}O_3$	154.16	3,4-Dihydroxyphenylethanol	123.0445; 122.0368	Ligustrum lucidum
11	8.736	$[M+H]^+$	268.1024	268.104	-6.0	$C_{10}H_{13}N_5O_4$	267.1	Adenosine	268.1027; 136.0573; 119.0312	Prunella vulgaris, Ligustrum lucidum
12	10.035	$[M-H]^-$	315.1078	315.1085	-2.2	$C_{14}H_{20}O_8$	316.12	Cimidahurinine	153.0549; 123.0447	Ligustrum lucidum
13	10.87	$[M-H]^-$	313.0935	313.0929	1.9	$C_{14}H_{18}O_8$	314.1	Salicylic acid methyl ester glucoside	151.0396; 150.0318; 123.0453	Ginseng

14	11.262	$[M-H]^-$	137.0241	137.0244	-2.2	$C_7H_6O_3$	138.03	3,4-Dihydroxybenzaldehyde	137.0234; 108.0211; 92.0263; 81.0343; 65.0040; 53.0396	Prunella Vulgaris
15	13.23	$[M+FA-H]^-$	345.1186	345.1191	-1.4	$C_{14}H_{20}O_7$	300.12	Salidroside	119.0341; 101.0255; 89.0242; 59.0136	Ligustrum lucidum
16	16.249	$[M-H]^-$	431.1546	431.1559	-3.0	$C_{19}H_{28}O_{11}$	432.42	Osmanthuside H	191.0555; 149.0449; 89.0242	Ligustrum lucidum
17	16.536	$[M-H]^-$	371.0971	371.0984	-3.5	$C_{16}H_{20}O_{10}$	372.11	Dihydro Isoferulic Acid 3-O-β-D-Glucuronide	371.0976; 249.0609; 121.0290	Prunella Vulgaris
18	19.629	$[M+FA-H]^-$	447.1543	447.1508	7.8	$C_{18}H_{26}O_{10}$	402.15	Benzyl β-primeveroside	401.1439; 269.1014; 161.0455	Ginseng
19	23.401	$[M-H]^-$	403.1243	403.1246	-0.7	$C_{17}H_{24}O_{11}$	404.13	8-epikingiside	371.0964; 241.0344; 179.0347; 59.0135	Ligustrum lucidum
20	24.376	$[M-H]^-$	521.1284	521.1242	8.1	$C_{24}H_{26}O_{13}$	522.14	Salviaflaside	359.0758; 323.0757; 179.0342; 161.0234; 135.0442	Prunella Vulgaris
21	24.629	$[M-H]^-$	593.1509	593.1512	-0.5	$C_{27}H_{30}O_{15}$	594.16	Nicotiflorin	593.1498; 473.1056; 383.0768; 353.0656; 297.0758	Prunella Vulgaris
22	25.342	$[M+NH_4]^+$	360.166	360.1653	1.9	$C_{16}H_{22}O_8$	342.13	Coniferin	205.0687; 187.0575; 127.0352	Ligustrum lucidum
23	26.561	$[M-H]^-$	359.0758	359.0772	-3.9	$C_{18}H_{16}O_8$	360.08	Rosmarinic acid	197.0444; 179.0339; 161.0234; 133.0286	Prunella Vulgaris
24	28.055	$[M-H]^-$	785.2493	785.251	-2.2	$C_{35}H_{46}O_{20}$	786.26	Echinacoside	785.2470; 623.2172; 161.0238	Ligustrum lucidum
25	28.153	$[M-H]^-$	569.1487	569.1512	-4.4	$C_{25}H_{30}O_{15}$	570.16	Oleuropeinic acid	569.1500; 389.0877; 363.1079; 331.0811; 151.0395	Ligustrum lucidum
26	30.778	$[M-H]^-$	477.0679	477.0675	0.8	$C_{21}H_{18}O_{13}$	478.36	Quercetin 3-O-glucuronide	301.0335; 255.0290; 151.0029	Prunella vulgaris, Saururus chinensis
27	30.837	$[M-H]^-$	609.1447	609.1461	-2.3	$C_{27}H_{30}O_{16}$	610.15	Quercetin-3-O-neohesperidoside	609.1437; 301.0306; 300.0265; 271.0247	Prunella Vulgaris
28	30.943	$[M-H]^-$	463.0869	463.0882	-2.8	$C_{21}H_{20}O_{12}$	464.1	Hyperoside	463.0863; 300.0259; 271.0234; 255.0291; 151.0035	Prunella vulgaris, Saururus chinensis,

										and guava leaves
29	31.004	[M-H]$^-$	701.2295	701.2298	-0.4	$C_{31}H_{42}O_{18}$	702.24	Neonuezhenide	701.2271; 469.1345; 315.1075	Ligustrum lucidum
30	31.338	[M-H]$^-$	553.1551	553.1563	-2.2	$C_{25}H_{30}O_{14}$	554.5	Ligustrosidic acid	347.1126; 209.0448; 183.0658	Ligustrum lucidum
31	31.525	[M-H]$^-$	463.0865	463.0882	-3.7	$C_{21}H_{20}O_{12}$	464.1	Isoquercitrin	463.0859; 300.0257; 271.0230; 255.0287; 151.0031	Prunella vulgaris, Saururus chinensis, and guava leaves
32	31.735	[M-H]$^-$	447.0919	447.0933	-3.1	$C_{21}H_{20}O_{11}$	448.1	Luteolin 7-O-glucoside	447.0927; 285.0387	Ligustrum lucidum
33	32.154	[M-H]$^-$	685.233	685.2349	-2.8	$C_{31}H_{42}O_{17}$	686.24	Nuezhenide	523.1810; 453.1389; 421.1494; 299.1122	Ligustrum lucidum
34	32.424	[M-H]$^-$	623.1972	623.1981	-1.4	$C_{29}H_{36}O_{15}$	624.21	Verbascoside	623.1950; 461.1651; 161.0239	Ligustrum lucidum
35	32.546	[M-H]$^-$	433.0767	433.0776	-2.1	$C_{20}H_{18}O_{11}$	434.08	Reynoutrin	433.0748; 301.0336; 300.0256; 271.0232	Guava leaves
36	33.134	[M-H]$^-$	433.0758	433.0776	-4.2	$C_{20}H_{18}O_{11}$	434.08	Guaijaverin	433.0746; 300.0256; 271.0230; 255.0286	Guava leaves
37	33.468	[M-H]$^-$	609.1442	609.1461	-3.1	$C_{27}H_{30}O_{16}$	610.15	Rutin	609.1417; 301.0333; 300.0253; 271.0235	Prunella vulgaris, Saururus chinensis
38	33.677	[M-H]$^-$	685.2316	685.2349	-4.8	$C_{31}H_{42}O_{17}$	686.24	Specnuezhenide	523.1790; 453.1376; 421.1482	Ligustrum lucidum
39	33.933	[M-H]$^-$	623.1969	623.1981	-1.9	$C_{29}H_{36}O_{15}$	624.21	Isoacteoside	179.0341; 161.0252	Ligustrum lucidum
40	33.997	[M-H]$^-$	433.0761	433.0776	-3.5	$C_{20}H_{18}O_{11}$	434.08	Avicularin	301.0337; 300.0256; 271.0235; 178.9980	Guava leaves
41	34.546	[M-H]$^-$	447.0919	447.0933	-3.1	$C_{21}H_{20}O_{11}$	448.1	Quercitrin	447.0914; 300.0261; 271.0237	Guava leaves
42	34.865	[M-H]$^-$	481.0973	481.0988	-3.1	$C_{21}H_{22}O_{13}$	464.1	Myrciaphenone B	481.0959; 313.0551; 167.0344	Guava leaves
43	36.207	[M-H]$^-$	685.2325	685.2349	-3.5	$C_{31}H_{42}O_{17}$	686.24	Isonuezhenide	523.1789; 453.1374; 299.1113; 89.0238	Ligustrum lucidum
44	37.449	[M-H]$^-$	539.1749	539.177	-3.9	$C_{25}H_{32}O_{13}$	540.18	Oleuropein	539.1765; 377.1240; 307.0816	Ligustrum lucidum
45	39.235	[M-H]$^-$	715.1295	715.1305	-1.4	$C_{36}H_{28}O_{16}$	716.14	DehydroSalvianolic acid B	715.1285; 535.0867; 311.0549; 179.0342	Prunella vulgaris
46	40.029	[M+FA-	845.4885	845.4904	-2.2	$C_{42}H_{72}O_{14}$	800.49	Ginsenoside Rg1	845.4874; 799.4825; 637.4307;	Ginseng

		H]$^-$							161.0454	
47	40.283	[M+FA-H]$^-$	991.5475	991.5483	-0.8	$C_{48}H_{82}O_{18}$	946.55	Ginsenoside Re	945.5413; 799.4877; 161.0461	Ginseng
48	41.093	[M-H]$^-$	571.1432	571.1457	-4.4	$C_{28}H_{28}O_{13}$	572.15	Guavinoside B	571.1406; 313.0540; 257.0798; 169.0129	Guava leaves
49	41.879	[M-H]$^-$	519.1489	519.1508	-3.7	$C_{25}H_{28}O_{12}$	520.16	6-O-Cinnamoyl-8-epikingisidic acid	519.1497; 227.0557; 161.0603; 147.0445; 121.0654	Ligustrum lucidum
50	42.124	[M-H]$^-$	285.0415	285.0405	3.5	$C_{15}H_{10}O_6$	286.05	Kaempferol	285.0396; 151.0027; 133.0294	Guava leaves
51	42.176	[M-H]$^-$	301.0343	301.0354	-3.7	$C_{15}H_{10}O_7$	302.04	Quercetin	301.0331; 151.0031; 121.0291; 83.0139; 65.0031	Prunella vulgaris, Guava leaves
52	42.58	[M-H]$^-$	1071.3544	1071.3562	-1.7	$C_{48}H_{64}O_{27}$	1072.36	Nuezhenoside G13	1071.3532; 771.2369; 685.2334; 523.1806; 299.1127	Ligustrum lucidum
53	44.02	[M-H]$^-$	1071.3536	1071.3562	-2.4	$C_{48}H_{64}O_{27}$	1072.36	Oleonuezhenide	1071.3537; 909.3027; 685.2329; 523.1788	Ligustrum lucidum
54	45.986	[M-H]$^-$	327.2167	327.2177	-3.1	$C_{18}H_{32}O_5$	328.23	Trihydroxy Octadecadienoic acid	327.2158; 291.1960; 229.1437; 211.1333; 171.1019	Prunella vulgaris, Ginseng
55	46.249	[M+FA-H]$^-$	845.4892	845.4904	-1.4	$C_{42}H_{72}O_{14}$	800.49	Ginsenoside Rf	799.4825; 637.4340; 475.3811; 161.0447	Ginseng
56	46.932	[M+FA-H]$^-$	1153.5983	1153.6011	-2.4	$C_{54}H_{92}O_{23}$	1108.6	Ginsenoside Rb1	1107.5966; 179.0692	Ginseng
57	47.127	[M-H]$^-$	329.2329	329.2328	0.3	$C_{18}H_{34}O_5$	330.24	Trihydroxyoctadecanoic acid	329.2308; 229.1427; 211.1337; 171.1015; 139.1121	Prunella vulgaris, Ginseng
58	47.195	[M+FA-H]$^-$	683.4364	683.4376	-1.8	$C_{36}H_{62}O_9$	638.44	Ginsenoside Rh1	683.4349; 637.4310; 475.3806; 161.0442	Ginseng
59	47.744	[M-H]$^-$	287.2219	287.2228	-3.1	$C_{16}H_{32}O_4$	288.23	Dioxy hexadecanoic acid	287.2212; 285.2049; 127.1119	Prunella vulgaris, Ginseng
60	47.941	[M+FA-H]$^-$	991.5525	991.5483	4.2	$C_{48}H_{82}O_{18}$	946.55	Ginsenoside Rd	945.5411; 783.4961; 663.3699	Ginseng
61	48.175	[M-H]$^-$	329.2326	329.2328	-0.6	$C_{18}H_{34}O_5$	330.24	Trihydroxyoctadecanoi	329.2320; 211.1334; 201.1124;	Prunella vulgaris,

								c acid	171.1028; 139.1105	Ginseng
62	49.875	$[M+H]^+$	345.1699	345.1697	0.6	$C_{20}H_{24}O_5$	344.16	Machilin D	221.1152; 165.0874; 151.0350; 137.0556	Saururus chinensis
63	50.812	$[M-H]^-$	487.3411	487.3429	-3.7	$C_{30}H_{48}O_5$	488.35	Asiatic acid	487.3398; 469.3277; 409.3089	Prunella vulgaris
64	52.032	$[M-H]^-$	269.0809	269.0819	-3.7	$C_{16}H_{14}O_4$	270.09	Pinostrobin	227.0708; 165.0189; 121.0290; 79.0187	Guava leaves
65	54.081	$[M-H]^-$	329.1747	329.1758	-3.3	$C_{20}H_{26}O_4$	330.18	(-)-Dihydroguaiaretic acid	314.1528; 121.0301	Saururus chinensis
66	54.545	$[M+H]^+$	357.1352	357.1333	5.3	$C_{20}H_{20}O_6$	356.13	Sauchinone	327.1235; 309.1131; 175.0360; 151.0357	Saururus chinensis
67	55.266	$[M+H]^+$	433.2218	433.2221	-0.7	$C_{24}H_{32}O_7$	432.51	Gelomulide N	265.1432; 247.1319; 216.1121; 169.0831	Saururus chinensis
68	55.887	$[M-H]^-$	325.1442	325.1445	-0.9	$C_{20}H_{22}O_4$	326.39	Licarin A	295.0977; 197.0294	Saururus chinensis
69	57.668	$[M+H]^+$	277.2163	277.2167	-1.4	$C_{18}H_{28}O_2$	276.21	12-phenyldodecanoic acid	277.2129; 179.1403; 135.1138	Saururus chinensis
