# Supplementary material for: TangNaikang Formula Alleviates Podocyte Injury in Diabetic Nephropathy by Modulating the SHIP2/PI3K/AKT Pathway
Source: J Diabetes Res. 2025 Nov 19;2025:6568591. doi: 10.1155/jdr/6568591 (PMC12657081; doi:10.1155/jdr/6568591)
Supplement: Supporting Information 3 — Table S3: UPLC-Q-TOF/MS analysis of TangNaikang-containing serum. [file 6568591.f3.pdf]

Table S3. UPLC-Q-TOFMS analysis of Tangnaikang-containing serum

Serial Number	Time (min)	Additive ion	*m/z* Actual value	*m/z* Theoretical value	ppm	Structural formula	Molecular weight	Components
P1	1.775	[M-H]-	191.0197	191.0197	0.0	$C_6H_8O_7$	192.12	Limonexic acid
P2	1.893	[M-H]-	169.0139	169.0142	-1.8	$C_7H_6O_5$	170.02	Gallic acid
M1	2.052	[M-H]-	232.9758	232.9761	-1.3	$C_7H_6O_7S$	233.98	Protocatechuic acid+sulfation
M2	2.06	[M-H]-	277.002	277.0024	-1.4	$C_9H_{10}O_8S$	246.02	Salvianic acid A +sulfation
P3	3.352	[M-H]-	197.0428	197.0455	-13.7	$C_9H_{10}O_5$	198.05	Salvianic acid A
M3	3.414	[M-H]-	313.0925	313.0929	-1.3	$C_{14}H_{18}O_8$	314.1	Salidroside+deglycosylation+glucuronidation
M4	3.51	[M-H]-	341.0872	341.0878	-1.8	$C_{15}H_{18}O_9$	342.1	Salvianic acid A + didehydroxylation + glucuronidation
M5	3.606	[M-H]-	261.0081	261.0071	3.8	$C_9H_{10}O_7S$	262.01	Salvianic acid A +Dehydroxylation +Sulfation
M6	3.626	[M-H]-	355.0663	355.0671	-2.3	$C_{15}H_{16}O_{10}$	356.07	Salvianic acid A +Dehydroxylation+glucuronidation
M7	3.77	[M-H]-	475.1447	475.1457	-2.1	$C_{20}H_{28}O_{13}$	476.15	Salidroside+glucuronidation
M8	4.146	[M-H]-	261.0085	261.0071	5.4	$C_9H_{10}O_7S$	262.01	Salvianic acid A +dehydroxylation+sulfation
M9	4.338	[M-H]-	387.0912	387.0933	-5.4	$C_{16}H_{20}O_{11}$	388.1	Salvianic acid A +Methylation+Glucuronidation
M10	4.434	[M-H]-	163.0399	163.0401	-1.2	$C_9H_8O_3$	164.05	Salvianic acid A +dehydration+dehydroxylation
M11	4.497	[M-H]-	193.0502	193.0506	-2.1	$C_{10}H_{10}O_4$	194.06	Salvianic acid A +methylation+dehydration
M12	4.549	[M-H]-	245.0119	245.0125	-2.4	$C_9H_{10}O_6S$	246.02	Salvianic acid A +didehydroxylation+sulfation

M13	4.712	[M-H]-	137.0244	137.0244	0.0	$C_7H_6O_3$	138.12	Gallic acid+Dehydroxylation+Dehydroxylation
M14	5.041	[M-H]-	165.0554	165.0557	-1.8	$C_9H_{10}O_3$	166.06	Salvianic acid A +Didehydroxylation
M15	5.125	[M-H]-	217.0169	217.0176	-3.2	$C_8H_{10}O_5S$	218.02	Salidroside+deglycosylation+sulfation
M16	5.125	[M-H]-	137.0607	137.0608	-0.7	$C_8H_{10}O_2$	138.16	Tyrosol
M17	5.822	[M-H]-	379.06997	379.0704	-1.1	$C_{14}H_{20}O_{10}S$	380.08	Salidroside+sulfation
M18	5.841	[M-H]-	165.0555	165.0557	-1.2	$C_9H_{10}O_3$	166.06	Salvianic acid A +Didehydroxylation
M19	7.981	[M-H]-	380.9898	380.9922	-6.3	$C_{15}H_{10}O_{10}S$	382	Quercetin+sulfation
M20	9.547	[M-H]-	395.0075	395.0073	0.5	$C_{16}H_{12}O_{10}S$	396.02	Quercetin+methylation+sulfation
M21	9.606	[M-H]-	315.049	315.051	-6.3	$C_{16}H_{12}O_7$	316.06	Quercetin+methylation
M22	9.934	[M-H]-	315.1086	315.1085	0.3	$C_{14}H_{20}O_8$	316.12	Salidroside+Hydroxymethylene loss
M23	13.119	[M-H]-	299.1133	299.1136	-1.0	$C_{14}H_{20}O_7$	300.12	Salvianic acid A +reduction+Glycation
P4	13.205	[M+FA-H]-	345.1188	345.1191	-0.9	$C_{14}H_{20}O_7$	300.12	Salidroside
M24	39.004	[M-H]-	359.0409	359.0409	0.0	$C_{17}H_{12}O_9$	360.27	Quercetin+Acetylation+oxidation
P5	43.135	[M-H]-	519.1488	519.1508	-3.9	$C_{25}H_{28}O_{12}$	520.16	6'-O-Cinnamoyl-8-epikingisidic acid
P6	44.812	[M-H]-	269.0811	269.0819	-3.0	$C_{16}H_{14}O_4$	270.09	Pinostrobin
P7	47.317	[M-H]-	285.0433	285.0405	9.8	$C_{15}H_{10}O_6$	286.05	Kaempferol
P8	48.584	[M-H]-	325.1449	325.1445	1.2	$C_{20}H_{22}O_4$	326.39	Licarin A
P9	50.868	[M+H]+	357.1358	357.1333	7.0	$C_{20}H_{20}O_6$	356.13	Sauchinone
M25	58.993	[M+H]+	477.3998	477.3938	12.6	$C_{30}H_{52}O_4$	476.39	Protopanaxatriol

Note: P: prototype constitutions prototype components, M: metabolite
